# A New Photoelectrocatalytic Water Purification System for Simultaneous Removal of Organic Pollutants and Heavy Metal Ions in Water

**DOI:** 10.34133/research.0654

**Published:** 2025-04-16

**Authors:** Peng Zhou, Yingming Xu, Tianhong Cui

**Affiliations:** Department of Mechanical Engineering, University of Minnesota, Minneapolis, MN 55455, USA.

## Abstract

A new photoelectrocatalytic water purification system was investigated by combining photocatalysis and electrochemistry. This configuration achieves simultaneous removal of both organic compounds and inorganic heavy metal ions from water by taking a carbon electrode as the working electrode and another electrode coated with a photocatalyst as the counter electrode. A negative bias potential is imparted onto the working electrode to induce the reduction of heavy metal ions, whereas the photocatalytic degradation of organic pollutants on the counter electrode is amplified via the transfer of photoexcited electrons from the counter electrode to the working electrode. Evaluations conducted in bulk solutions demonstrated that photoelectrocatalysis surpassed photocatalysis by yielding an organic matter degradation efficiency 2.3 times higher, successfully degrading 98% of a 10 μM methylene blue solution within 2 h. Simultaneously, the system realized the recovery of heavy metal ions, including copper, lead, and cadmium. This new photoelectrocatalytic water purification system was further integrated with microchannels, and the testing data affirm the substantial potential for system miniaturization.

## Introduction

Water scarcity and pollution are substantial global challenges that have important implications for sustainable ecosystems. Currently, over 2 billion people worldwide lack access to sufficient clean water for their basic needs, such as drinking, cooking, and sanitation, and the demand for clean water is expected to increase by 50% by 2050 due to population growth and economic development [1]. However, water pollution exacerbates the situation. According to a global survey by the Food and Agriculture Organization of the United Nations, over 80% of wastewater is discharged into the environment without proper treatment, leading to 800,000 premature deaths in 2012 [2]. Water pollution affects over 2 billion people worldwide, causing a range of health problems such as diarrhea, cholera, and typhoid fever [3,4]. Therefore, a low-cost and efficient water treatment technology is of importance for the sustainable development of water resources.

Polluted water contains various contaminants, including suspended solids, organic matter, nutrients, pathogens, metals, and inorganic dissolved matter [5]. Conventional technologies for water purification, such as adsorption, coagulation, sedimentation, chemical filtration, and membrane filtration, are relatively inefficient and generate toxic secondary pollution [6–8]. Photocatalytic water treatment based on photocatalysts is a promising low-cost sustainable water treatment technology. This technology uses electron–hole pairs generated by photocatalysts under light illumination to produce active radicals that degrade organic pollutants in water [9,10]. However, its efficiency is limited by the recombination of photoexcited electrons and holes, which reduces the lifetime of photogenerated holes [11].

Numerous methods have been developed to prevent the recombination of electrons and holes in photocatalysts. For example, heterostructured photocatalysts prevent electron–hole recombination by transferring photoexcited electrons from a semiconductor with a higher conduct band minimum to one with a lower conduct band minimum. The TiO_2_/CdS system was the first heterostructured photocatalyst designed by Spanhel et al. [12] in 1987. A large amount of research has attempted to improve photocatalytic efficiency by doping metals [13,14], metal oxides [15], nonmetals [16], or graphene [17–19] onto photocatalysts to transfer photogenerated electrons. However, these methods involve transferring electrons to a lower energy level, leading to the release of a portion of the potential energy of the transferred electrons and a reduced redox potential, which limits the scope of photocatalysis applications.

Photoelectrocatalysis (PEC) is considered an effective method to address the recombination of electron–hole pairs in photocatalysts. PEC applies a bias potential to a photocatalyst to induce electron migration from the photocatalyst to another electrode, thereby achieving the separation of electron–hole pairs and enhancing the efficiency of photocatalysis. Studies have reported that applying a bias potential as low as 0.1 V can achieve 2.59 times higher photodegradation efficiency [20]. Another important benefit of PEC is the removal of heavy metal ions at the cathode due to the reduction ability of electrons in the counter electrode [21–23]. However, the heavy metal ions that can be reduced at the cathode are limited [24]. In a titanium dioxide (TiO_2_)-based PEC system, although published works have shown that heavy metal ions with a high redox potential, such as copper and lead, can be reduced by photogenerated electrons. The potential of these electrons is generally considered insufficient to reduce heavy metal ions with a lower redox potential, such as cadmium [25].

Therefore, this study presents a new configuration of a 3-electrode PEC water purification system that is distinct from the existing setups. This approach achieves the reduction of all heavy metal ions and the efficiency enhancement of photocatalytic organic degradation.

## Results

### Characterization and optimization of photocatalyst electrodes

**Fig. 1.**
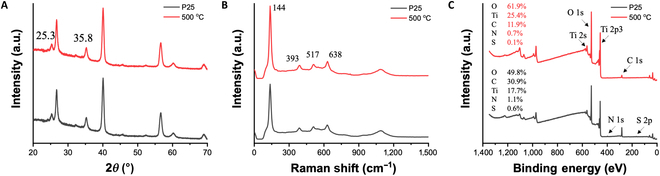
Characterization of the photocatalyst electrode before and after annealing at 500 °C: (A) x-ray diffraction (XRD) patterns, (B) Raman spectra, and (C) x-ray photoelectron spectroscopy (XPS) spectral analysis.

The structural and chemical properties of the photocatalyst electrodes before and after annealing at 500 °C were characterized using x-ray diffraction, Raman spectroscopy, and x-ray photoelectron spectroscopy, as shown in Fig. 1.

X-ray diffraction analysis (Fig. 1A) reveals that the TiO_2_ layer primarily exists in the anatase phase. The characteristic peaks of anatase TiO_2_ are weak, likely due to the thin layer of TiO_2_ deposited via the layer-by-layer (LBL) self-assembly process, causing the signal to be overshadowed by the strong peaks from the fluorine-doped tin oxide (FTO) substrate. Notably, there is no observable transformation to the rutile phase after annealing at 500 °C, as no new rutile-specific diffraction peaks emerge. This observation aligns with literature reports that indicate that the anatase-to-rutile phase transition typically occurs at temperatures between 600 and 700 °C [26].

Further confirmation of the anatase phase was obtained from Raman spectroscopy (Fig. 1B). The Raman spectra display 4 characteristic anatase peaks at 144, 393, 517, and 638 cm^−1^. These peaks remain nearly unchanged after annealing, reinforcing the conclusion that the crystal phase of TiO_2_ did not transition to rutile within the experimental temperature range.

The decomposition of the polyelectrolyte layers during annealing was verified using x-ray photoelectron spectroscopy (Fig. 1C). The reduction in the carbon content from 30.9% before annealing to 11.9% after annealing indicates the decomposition of poly(sodium 4-styrenesulfonate) (PSS). Additionally, the reduction of nitrogen and sulfur signals suggests the removal of the polyelectrolyte layers, leading to a more compact TiO_2_ nanoparticle network.

**Fig. 2.**
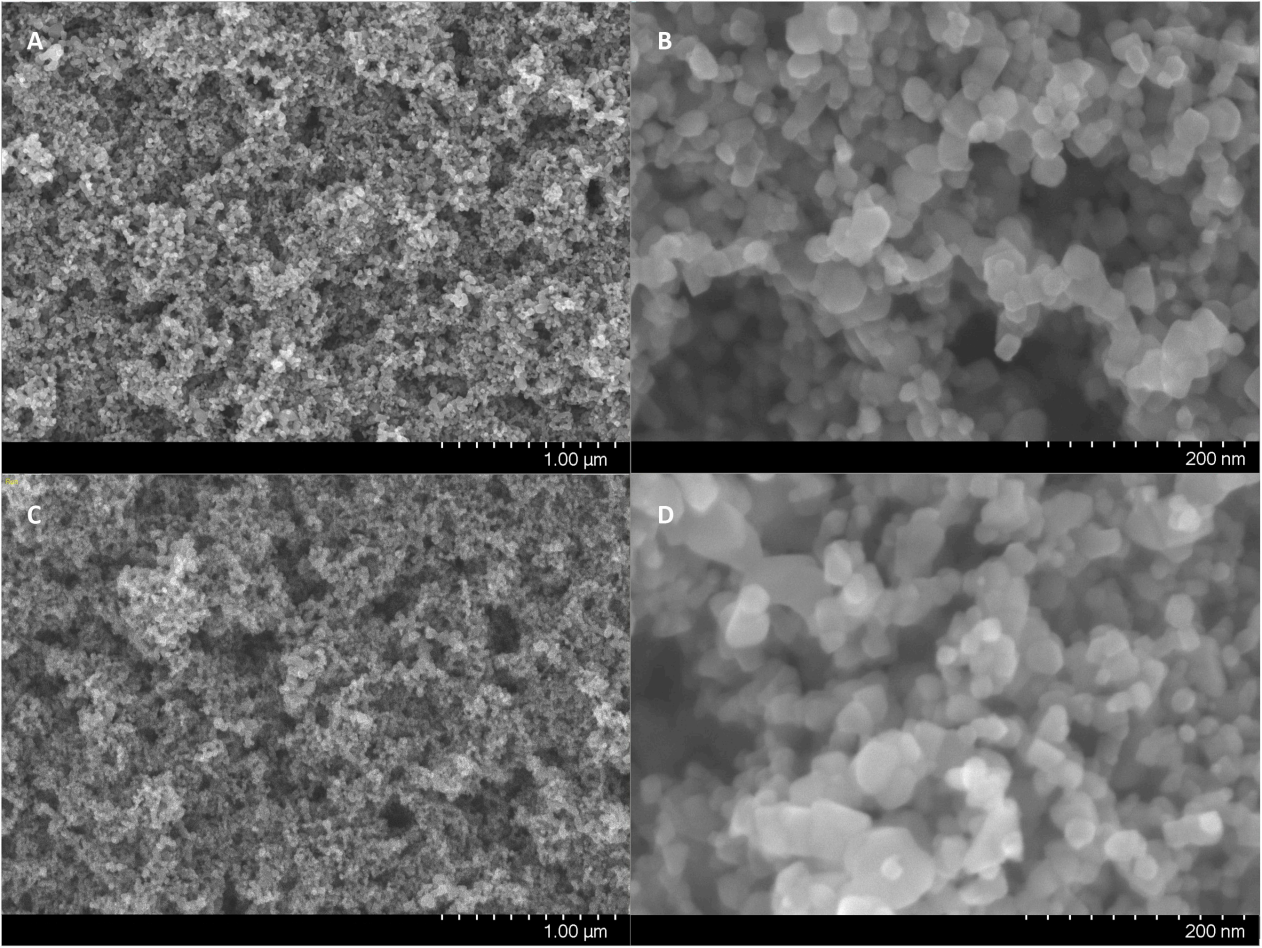
Scanning electron microscopy (SEM) images of photocatalyst before and after 500 °C annealing: (A) 8 poly(sodium 4-styrenesulfonate) (PSS)/TiO_2_ bilayers on a glass substrate before annealing, at low resolution; (B) 8 PSS/TiO_2_ bilayers on a glass substrate before annealing, at high resolution; (C) 8 PSS/TiO_2_ bilayers on a glass substrate after annealing, at low resolution; (D) 8 PSS/TiO_2_ bilayers on a glass substrate after annealing, at high resolution.

The morphology of immobilized photocatalysts before and after annealing was observed by scanning electron microscopy (SEM). Figure 2A and B shows the SEM images of 8 PSS/TiO_2_ bilayers on an FTO glass before annealing with different resolutions, while Fig. 2C and D shows the SEM images of the same sample after 500 °C annealing. The TiO_2_ nanoparticles can be clearly seen in the high-resolution image, and the size of the nanoparticles are about 25 nm in diameter. By comparing the SEM images before and after annealing, it can be clearly seen that the pores in the porous structure formed by TiO_2_ nanoparticles are reduced after annealing, indicating that the particles become closer to each other. This is due to the decomposition of poly(diallyl dimethyl ammonium chloride) (PDDA) and PSS layers and the reorganization of the arrangement of TiO_2_ nanoparticles at high temperatures. The benefit of this change is that the binding between TiO_2_ and the substrate becomes tighter, and the electron transfer between titanium dioxide nanoparticles and the substrate is faster. Therefore, a higher photocurrent and more efficient electron transfer can be expected in the PEC.

**Fig. 3.**
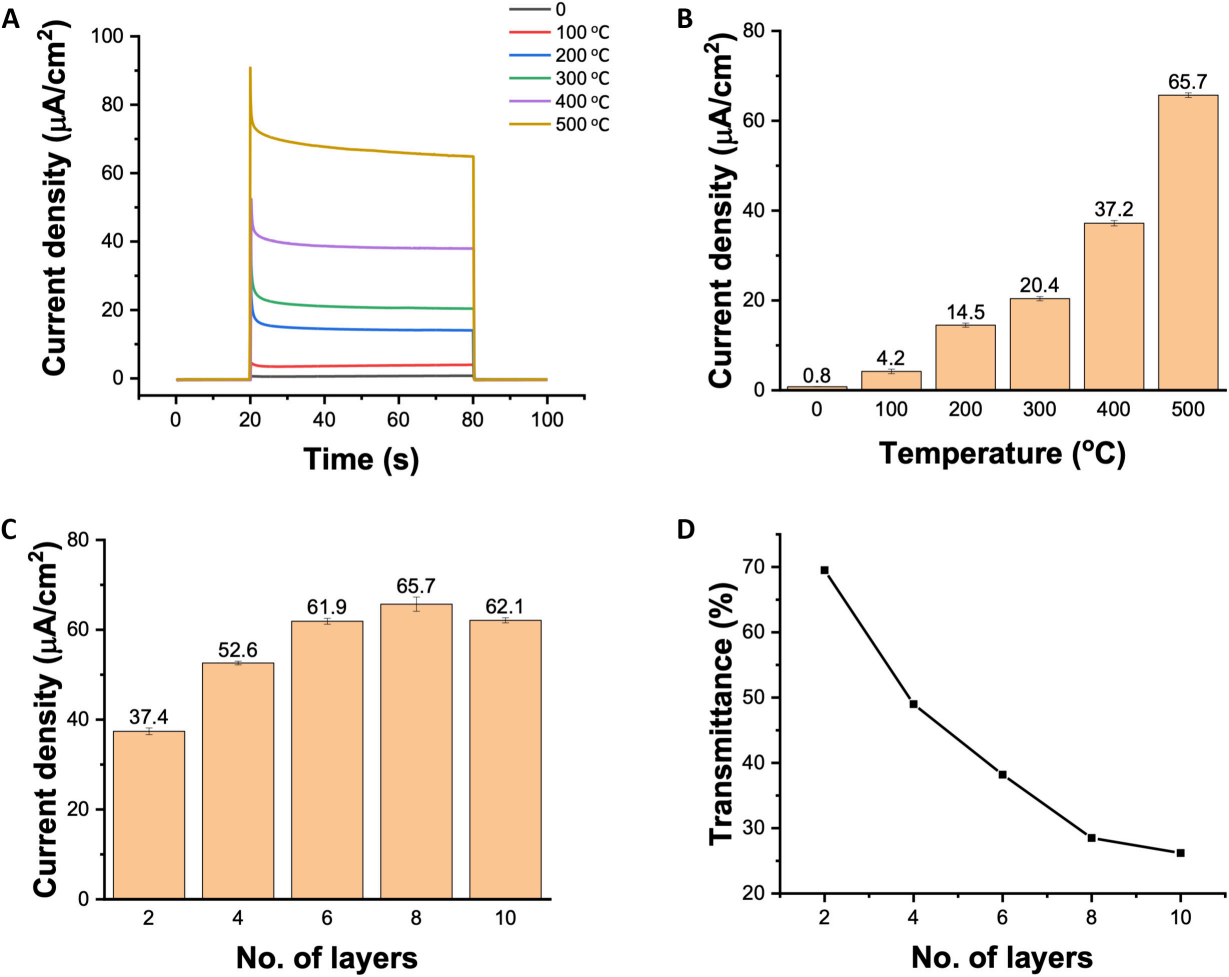
(A) Comparison of the photoelectrochemical response of photocatalyst electrodes at different annealing temperatures. (B) Comparison of the photocurrent generated by photocatalyst electrodes at different annealing temperatures. (C) Comparison of the photocurrent generated by photocatalyst electrodes with different bilayers of PSS/TiO_2_. (D) Transmittance of 365-nm ultraviolet light through photocatalyst electrodes with different bilayers of PSS/TiO_2_.

This consumption can be confirmed by measuring the photocurrent in photoelectrochemistry. Figure 3A illustrates the photoelectrochemical response of the photocatalyst electrodes prepared at different annealing temperatures. All of the photocatalyst electrodes were coated with 8 bilayers of PSS/TiO_2_ via the LBL self-assembly process prior to annealing. The potential difference between the photocatalytic electrode and the reference electrode was maintained at 0 V. The photocatalytic electrodes were first stabilized in the dark for 20 s. Upon opening the solar light simulator, all of the electrodes exhibited a photocurrent, which rapidly dropped to the background current when turning off the light. A comparison, as shown in Fig. 3B, reveals that the photocurrent density increases with increasing annealing temperatures. Specifically, the electrode annealed at 500 °C for 5 h exhibited a photocurrent 82 times higher than that of the nonannealed electrode, reaching 65.7 μA/cm^2^. This improvement can be attributed to the decomposition of PDDA and PSS and the rearrangement of TiO_2_ nanoparticles at high temperatures.

The optimization of the number of PSS/TiO_2_ bilayers coated on the photocatalyst electrode via the LBL self-assembly process was also evaluated through photoelectrochemical testing. As shown in Fig. 3C, the photocurrent density increases as the number of PSS/TiO_2_ bilayers gradually increases from 2 to 8. This is attributed to the larger coverage of TiO_2_ on the electrode surface, resulting in the generation of more photoexcited electrons. However, when the number of PSS/TiO_2_ bilayers exceeds 8, the photocurrent density tends to saturate and even exhibit a decrease. This phenomenon can be explained by the results, as shown in Fig. 3D, demonstrating the transparency of the prepared photocatalytic electrodes under 365-nm ultraviolet (UV) light. A higher number of PSS/TiO_2_ bilayers leads to lower transparency, indicating the reduction in the light intensity received by the underlying layers of the photocatalyst. In addition, the electrons generated by the surface-layer photocatalyst require a longer pathway to reach the FTO surface. Therefore, a decrease in photocurrent density is observed when the number of PSS/TiO_2_ bilayers exceeds 8. Ultimately, 8 PSS/TiO_2_ bilayers were selected as the optimized configuration for the photocatalyst electrode.

### PEC water purification system

In traditional PEC water treatment systems, a photocatalyst electrode is typically used as the anode to degrade organic pollutants by using photogenerated holes. Another metal electrode serves as the cathode, where the transferred photon-excited electrons are utilized to reduce heavy metal ions. The ultimate purpose of PEC is to separate the electron–hole pairs. Therefore, when combining a photocatalyst electrode with an electrochemical 3-electrode system, there are 2 different configurations. One commonly used configuration, as mentioned earlier regarding photoelectrochemical testing, involves using the photocatalyst electrode as the working electrode, where oxidation reactions occur to remove organic pollutants from water. The other electrode (in this work, a graphite rod) is used as the counter electrode, where photogenerated electrons are transferred to undergo reduction reactions, reducing heavy metal ions from the water. The roles of the photocatalyst electrode and carbon rod are reversed in the second configuration; a negative potential is applied on the graphite rod working electrode to reduce heavy metal ions. The photocatalyst electrode works as the counter electrode, still generating oxidation reactions through photogenerated holes. Both configurations enable the transfer of photogenerated electrons from the photocatalyst electrode to the graphite rod electrode. The following experiments compare the difference between the 2 configurations in terms of organic pollutant degradation and heavy metal ion reduction.

**Fig. 4.**
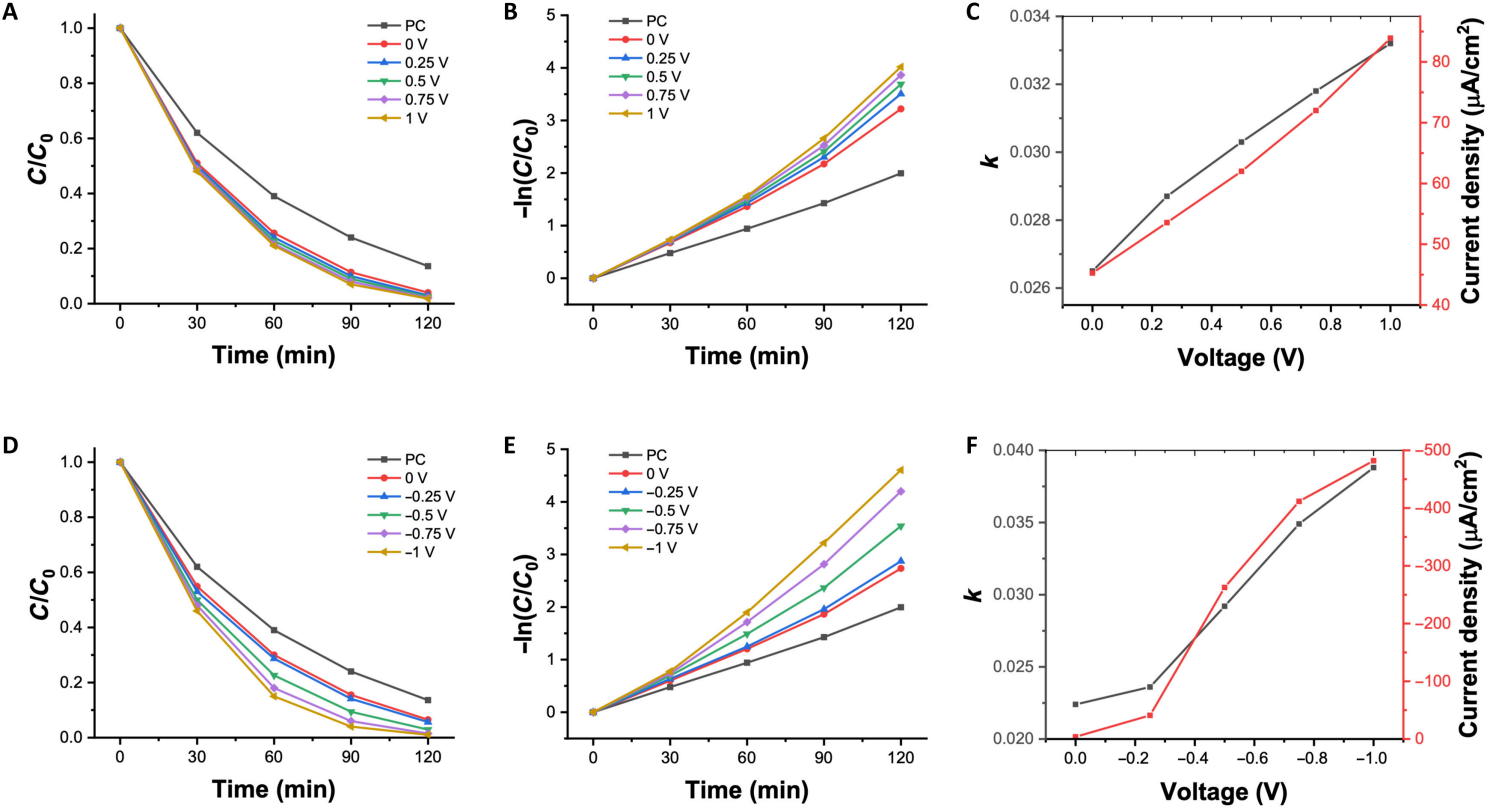
Relative methylene blue (MB) concentration versus irradiation time at different bias potentials in the (A) traditional photoelectrocatalysis (PEC) configuration and (D) new PEC configuration. MB degradation kinetics at different bias potentials in the (B) traditional PEC configuration and (E) new PEC configuration. Relationship between photodegradation constant k, current density, and bias potential in the (C) traditional PEC configuration and (F) new PEC configuration. PC, photocatalysis.

The efficiency of PEC systems for the degradation of organic compounds was evaluated through the degradation of methylene blue (MB) solutions. The MB degradation was tested at different electrode potentials in 2 configurations, and the concentration change is shown in Fig. 4A and D. The concentration of MB continuously decreases with increasing illumination time. The lower the MB concentration, the slower the concentration change rate, and the photodegradation kinetics of MB can be described by a first-order kinetic equation [27]:

$$\text{ln}\left(C/C_{0}\right)=-\mathrm{kt}$$ (1)

where C and $C_{0}$ are the MB concentrations at the beginning and during light illumination, respectively; t is time; and k is the reaction rate constant, which can be used to represent the photodegradation efficiency. Therefore, the slopes in Fig. 4B and E indicate the photodegradation efficiency corresponding to the 2 configurations.

Pure photocatalysis was tested for comparison to demonstrate the enhancement of the photodegradation efficiency of organic compounds using the 2 different PEC systems. When the photocatalyst electrode serves as the working electrode, as shown in Fig. 4A and B, the PEC system displays a 2-fold increase in photodegradation efficiency, compared to pure photocatalysis at a working electrode potential of 1.0 V. When the graphite rod serves as the working electrode, as shown in Fig. 4D and E, the PEC system exhibits a maximum photodegradation efficiency 2.3 times higher than that of pure photocatalysis. When the photocatalyst electrode serves as the working electrode, a marked improvement in photocatalytic degradation efficiency can be achieved even at a relatively low voltage, whereas in the other configuration, the enhancement of photocatalytic efficiency is clearly dependent on the working electrode potential. This is due to the different variations in the current magnitude with respect to the working electrode potential in different configurations, as shown in Fig. 4C and F. When the photocatalyst electrode serves as the working electrode, the current in the system originates from the photocurrent and the redox current at the working electrode. Since the photocurrent is much larger than the redox current and not affected by the electrode potential, substantial currents can be generated at low electrode potentials, enabling the efficient separation of photon-excited electron–hole pairs, leading to a notable improvement in photocatalytic degradation efficiency. On the other hand, when the graphite rod serves as the working electrode, the current in the PEC system is determined mainly by the heavy metal ions’ reduction current on the graphite rod electrode. This current magnitude is dependent on the electrode potential. When the electrode potential becomes more negative (larger in absolute values), the reduction reaction on the electrode becomes more vigorous, resulting in a larger current. When the consumption rate of electrons on the working electrode is lower than the rate of photogenerated electron production on the photocatalyst electrode, only a partial separation of photon-excited electron–hole pairs occurs, leading to a smaller improvement in photocatalytic degradation efficiency. However, when the potential on the working electrode becomes negative enough to generate an electron consumption rate exceeding the number of photogenerated electrons, the counter electrode potential rapidly increases to facilitate additional electrochemical degradation of organic compounds, generating sufficient electrons. Therefore, a higher enhancement in photocatalytic degradation rates can be observed compared to that of the previous configuration.

**Fig. 5.**
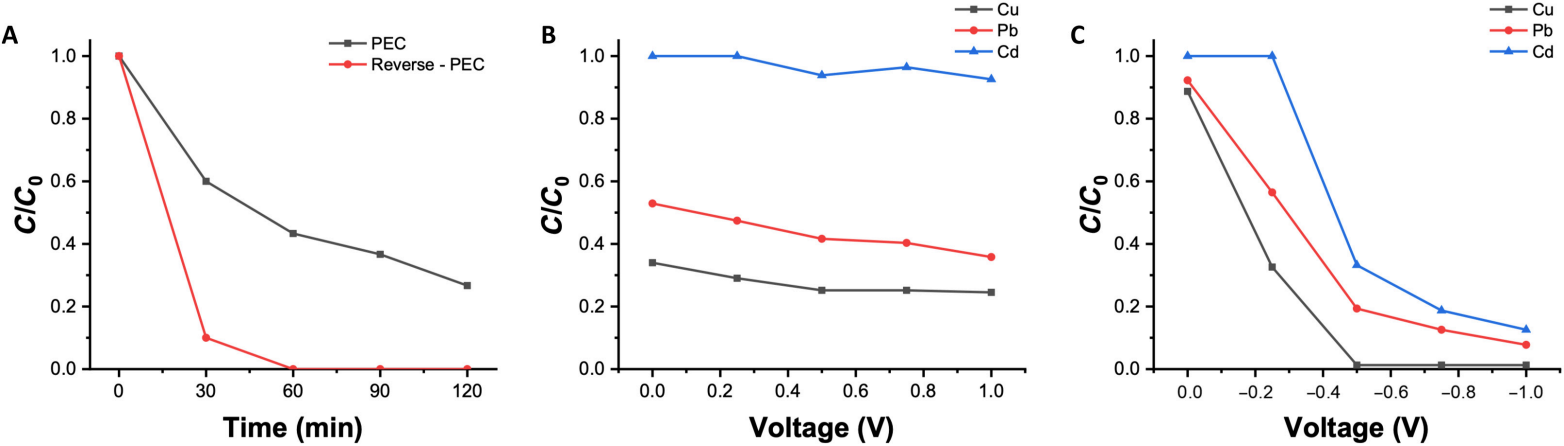
(A) Relative Cu^2+^ concentration versus irradiation time with different PEC configurations. (B) Relative heavy metal ions’ (Cu^2+^, Pb^2+^, and Cd^2+^) concentrations after 2-h reduction in PEC with the traditional configuration at different applied voltages. (C) Relative heavy metal ions’ (Cu^2+^, Pb^2+^, and Cd^2+^) concentrations after 2-h reduction in PEC with the new configuration at different applied voltages.

The 2 different configurations of the PEC system exhibit more notable differences in the reduction of heavy metal ions. Figure 5A records the reduction of copper ions (Cu^2+^) in the 2 PEC system configurations when the absolute value of the working electrode potential is 1.0 V. In the new PEC system with the graphite rod as the working electrode, the concentration of Cu^2+^ rapidly decreases and is completely recovered within 1 h. In contrast, when the photocatalyst electrode serves as the working electrode, only 72% of Cu^2+^ is recovered within 2 h. This phenomenon can also be explained by the current changes in Fig. 4C and F. When the absolute value of the working electrode potential is 1.0 V, the current density in the new PEC system with the graphite rod as the working electrode is much higher than the current density when the photocatalyst electrode serves as the working electrode, and a higher current density implies a faster reduction of heavy metal ions.

The reduction effects of different heavy metal ions were compared in the 2 different configurations of PEC systems. In addition to Cu^2+^, the reduction of lead (Pb^2+^) and cadmium (Cd^2+^) ions was also tested. Figure 5B and C shows the concentrations of heavy metal ions in the solution operation of the PEC systems for 2 h. The traditional configuration of the PEC system cannot reduce Cd^2+^, and its reduction efficiency for Cu^2+^ and Pb^2+^ is also low, with no marked changes observed with increasing electrode potential. This is because the number of photogenerated electrons is limited, and the absolute value of the photogenerated electron potential is much lower than the redox potential of Cd^2+^. In the new PEC system, reduction of all 3 heavy metal ions can be observed, and the efficiency of heavy metal ion reduction increases with a more negative electrode potential. More importantly, reduction of Cd^2+^ can be observed when the working electrode potential is lower than the redox potential of Cd^2+^, which cannot be achieved with the traditional configuration of the PEC system.

Overall, compared to the traditional PEC configuration, the new system with the graphite rod as the working electrode demonstrates notable advantages in both organic pollutant degradation and heavy metal ion reduction. In terms of pollutant degradation, this configuration enhances the separation of photogenerated electron–hole pairs by utilizing the working electrode potential to regulate electron consumption, thereby improving photocatalytic efficiency. In contrast, the traditional system relies primarily on photocurrent, limiting its ability to further enhance performance through potential control. Regarding heavy metal ion reduction and recovery, the new PEC configuration optimizes electron utilization by transferring photogenerated electrons to the graphite rod, creating a dedicated reduction zone. This ensures a more sufficient electron supply and enables the application of more negative potentials, facilitating the efficient reduction of a broader range of heavy metal ions, including those with lower reduction potentials.

### PEC microfluidic water purification system

Finally, the proposed PEC configuration was integrated with a microfluidic system to form a miniaturized, on-chip, efficient PEC water treatment system. The proposed new PEC configuration was implemented during testing, where the photocatalyst electrode served as the counter electrode and the Cu electrode served as the working electrode. A mixed solution containing the organic compound MB and inorganic heavy metal ions was passed through the microchannel at flow rates of 5, 7.5, and 10 ml/h. The concentrations of MB and heavy metal ions at the outlet were collected and measured. To prevent MB reduction at the working electrode and ensure accurate assessment of photocatalytic degradation efficiency, the solution was stirred overnight to oxidize reduced MB.

**Fig. 6.**
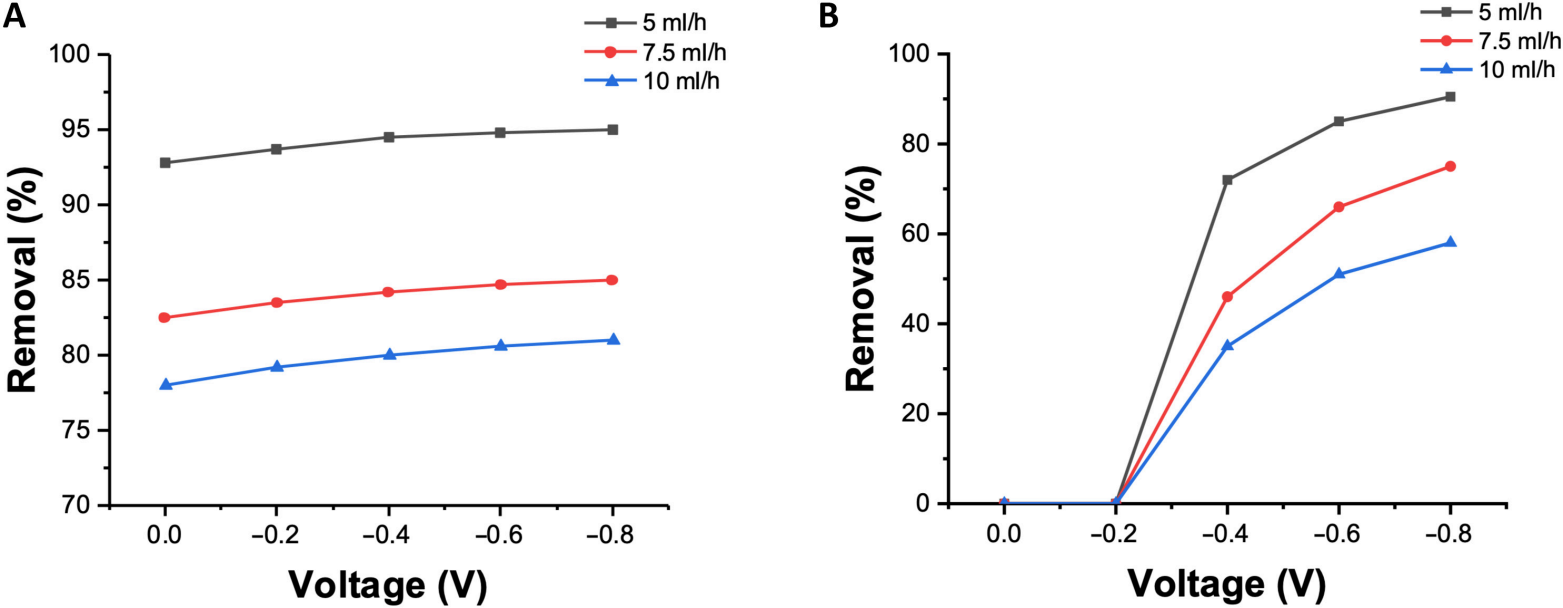
Performance of a microfluidic PEC system with different mass flow rates under different applied potentials on (A) MB removal and (B) heavy metal ions’ removal (Cd^2+^).

Figure 6A illustrates the efficiency of the microfluidic PEC system in degrading the organic compound MB at different flow rates when the potential of the working electrode decreases from 0 to −0.8 V. The results indicate an inverse relationship between the removal rate of MB and the flow rate. This phenomenon can be attributed to the shorter residence time of the solution in the microchannel at higher flow rates, leading to a shorter duration of the degradation reaction. Additionally, as the potential of the working electrode decreases, the removal rate of the organic compound increases. This can be explained by the acceleration of the reduction reaction on the working electrode at lower potentials, which enhances the electron transfer rate and facilitates efficient charge separation of photon-excited electrons and holes. Similarly, Fig. 6B demonstrates the efficiency of the microfluidic PEC system in recovering heavy metal ions. The effect of the flow rate on the reduction efficiency is similar to that of organic compound degradation. However, the working electrode potential has a more important influence on the efficiency of heavy metal ion recovery. Potentials higher than −0.2 V are insufficient for the reduction of Cd^2+^, whereas a decrease in potential below −0.4 V leads to a rapid increase in recovery efficiency. When the potential of the working electrode is set at −0.8 V, the system achieves over 95% degradation of 10 μM MB solution and over 90% degradation of a Cd ion solution with a concentration of 1 ppm at a flow rate of 5 ml/h.

This miniaturized microfluidic PEC system not only demonstrates high water treatment efficiency but also offers advantages such as integration, portability, and scalability, making it highly promising for on-site rapid water quality monitoring, portable water purification devices, and small-scale pollutant removal studies in laboratories. Furthermore, the design concept of this system can be extended for the modular development of large-scale PEC water treatment systems, enabling adaptable solutions for various water pollution treatment scenarios.

## Conclusion

This study presents a comprehensive investigation into the enhancement of water treatment efficiency through PEC compared to photocatalysis. Compared to existing PEC water treatment configurations, the new PEC system enables the simultaneous removal of organic compounds and most heavy metal ions. Both PEC system configurations demonstrate substantial improvements in terms of organic compound degradation and heavy metal ion recovery efficiency compared to photocatalysis. Notably, the proposed new configuration achieves an enhancement of 2.3 times in the degradation rate of organic compounds at a voltage of −1.0 V. Both configurations exhibit the recovery of Cu^2+^ and Pb^2+^ ions, but the proposed new configuration exhibits markedly higher recovery efficiency and is the only one capable of recovering all Cu^2+^, Pb^2+^, and Cd^2+^ ions. Furthermore, this study demonstrates the successful integration of the proposed PEC configuration into a miniaturized microfluidic system, achieving over 95% degradation of organic pollutants and more than 90% removal of heavy metal ions. This microfluidic PEC system not only provides an efficient and compact water treatment solution but also offers integration, portability, and scalability, making it highly suitable for on-site rapid water quality monitoring, portable water purification devices, and small-scale pollutant removal applications in laboratories. Additionally, the design concept of this system can be extended for the modular development of large-scale PEC water treatment systems, enabling adaptable solutions for various water pollution treatment scenarios. Overall, this study provides a theoretical foundation and model validation for both miniaturized PEC systems and high-efficiency large-scale PEC water treatment platforms, paving the way for future advancements in sustainable and effective water purification technologies.

## Materials and Methods

### Materials and reagents

Hydrochloric acid (HCl); sodium sulfate (Na_2_SO_4_); MB; Cu, Pb, and Cd standard solutions; PSS (Mw = 70,000); PDDA (20 wt% in water); and TiO_2_ nanoparticles (P25, 30% rutile and 70% anatase phases) were obtained from Sigma-Aldrich (St. Louis, MO, United States). All solutions were prepared with deionized water from an AmeriWater silex deionization system (Dayton, OH, United States).

### Device fabrication

Two types of devices were designed and fabricated for different experiments. One of them used LBL self-assembly to deposit TiO_2_ on a 2 cm × 2 cm FTO glass as the photocatalyst electrode. As described in another paper [28], 1 g of TiO_2_ was dissolved in 100 ml of HCl solution with pH of 3 to form a stable suspension. Then, the cleaned FTO glass was immersed in a solution containing positively charged conductive polyelectrolyte PDDA to change its surface charges. The FTO glass with a positively charged surface was then alternately immersed in PSS and TiO_2_ solutions for 10 min each, followed by rinsing with deionized water to remove unbound molecules after each deposition. Photocatalyst electrodes with different PSS/TiO_2_ bilayers were prepared by controlling the number of LBL self-assembly cycles. The electrode with the deposited photocatalyst was then placed in a furnace (MTI OTF-1200X-5L) and annealed at different temperatures. The heating rate was 2 °C/min, and the final annealing temperature was maintained for 5 h.

**Fig. 7.**
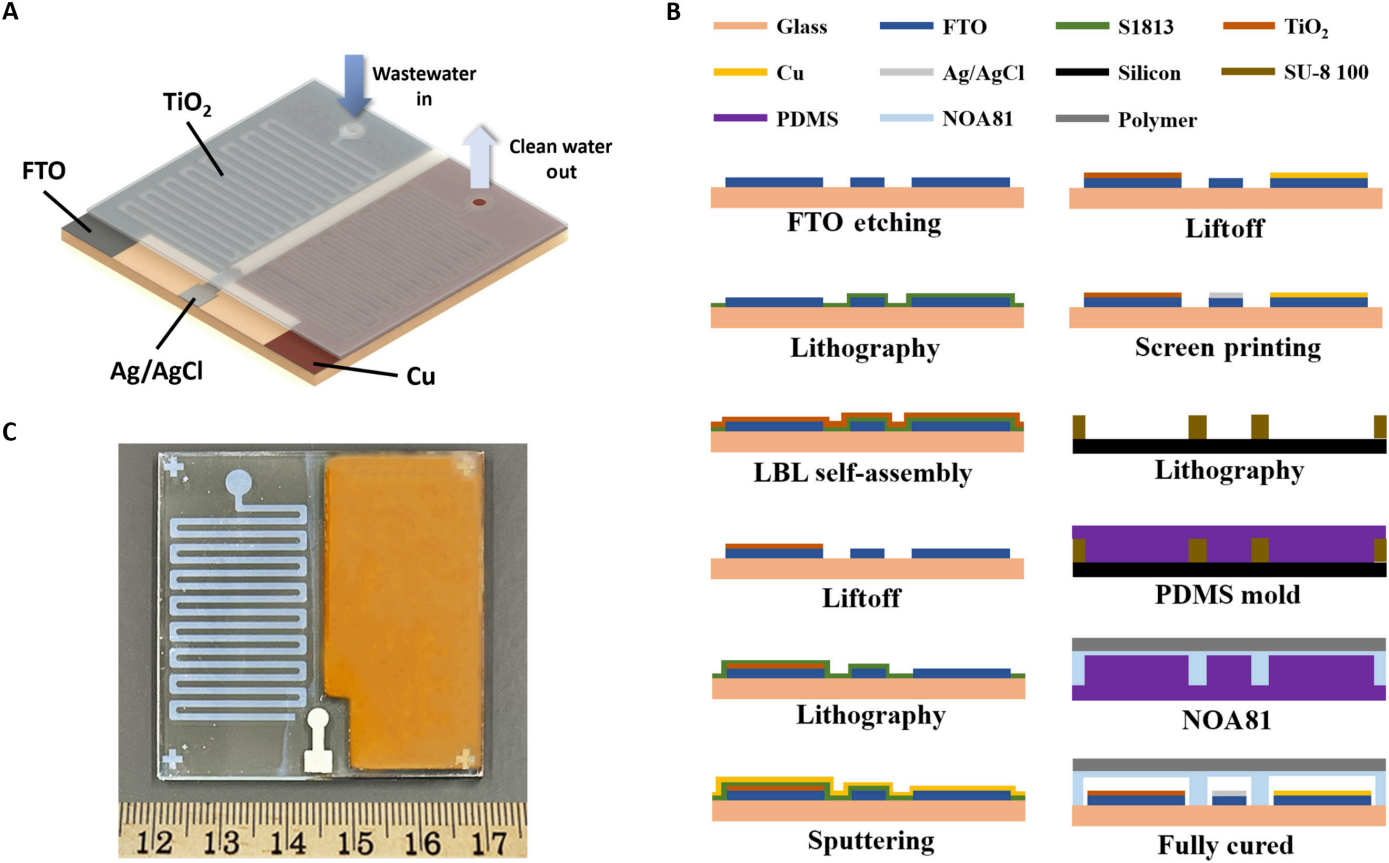
(A) Sketch of a microfluidic PEC water purification system. (B) Flowchart of the fabrication process. (C) Optical image of a fabricated PEC system on a chip. PDMS, polydimethylsiloxane; FTO, fluorine-doped tin oxide; LBL, layer-by-layer.

The integrated microfluidic PEC system was fabricated on a 5 cm × 5 cm FTO glass. The design and fabrication process of the proposed PEC system are illustrated in Fig. 7A and B, respectively. Firstly, the FTO glass was etched into the desired shape of the 3 electrodes using zinc and HCl [29]. The photocatalyst with the microchannel shape was deposited on the photocatalyst electrode using a liftoff process. Initially, a positive photoresist was used to pattern the microchannels on the FTO glass, and TiO_2_ was deposited using the LBL self-assembly method as described earlier. The photoresist was then removed by immersing the FTO glass in acetone followed by annealing in a furnace. The electrode for heavy metal ion reduction was deposited with a copper film 100 nm thick by sputtering to enhance the binding strength with the reduced heavy metal. A silver/silver chloride reference electrode was fabricated by screen printing of silver/silver chloride paste on the designed area. Figure 7C shows an optical image of the fabricated electrodes on FTO glass.

The microchannels were fabricated based on polydimethylsiloxane (PDMS) soft lithography [30]. The microchannel structures were first patterned with photolithography on a silicon wafer using negative photoresist SU-8 (100 μm thick). Next, a mold was created by pouring PDMS onto the silicon-based mold and curing it. The UV-curing glue (NOA81) was then applied on the PDMS mold with matching structures and covered by a UV-transparent polystyrene polymer film. After partially curing the glue under UV illumination, the PDMS mold was replaced with the FTO glass, and more UV illumination was applied to fully cure the glue.

### Measurement and characterization

The surface morphology of the photocatalyst electrode was observed using a field emission gun scanning electron microscope (Hitachi SU8230). Iridium (2.5 nm thick) was deposited on each sample to increase conductivity. The morphology of the deposited TiO_2_ was mainly compared before and after annealing.

The photoelectrochemical response of the fabricated photocatalyst electrode was evaluated by measuring the photocurrent produced in 0.1 M sodium sulfate solution. A solar simulator was used as the light source, and an electrochemical workstation was used to measure the photocurrent. The 2 cm × 2 cm photocatalyst electrode coated with TiO_2_ was used as the working electrode, a graphite rod electrode was used as the counter electrode, and an Ag/AgCl electrode was used as the reference electrode.

**Fig. 8.**
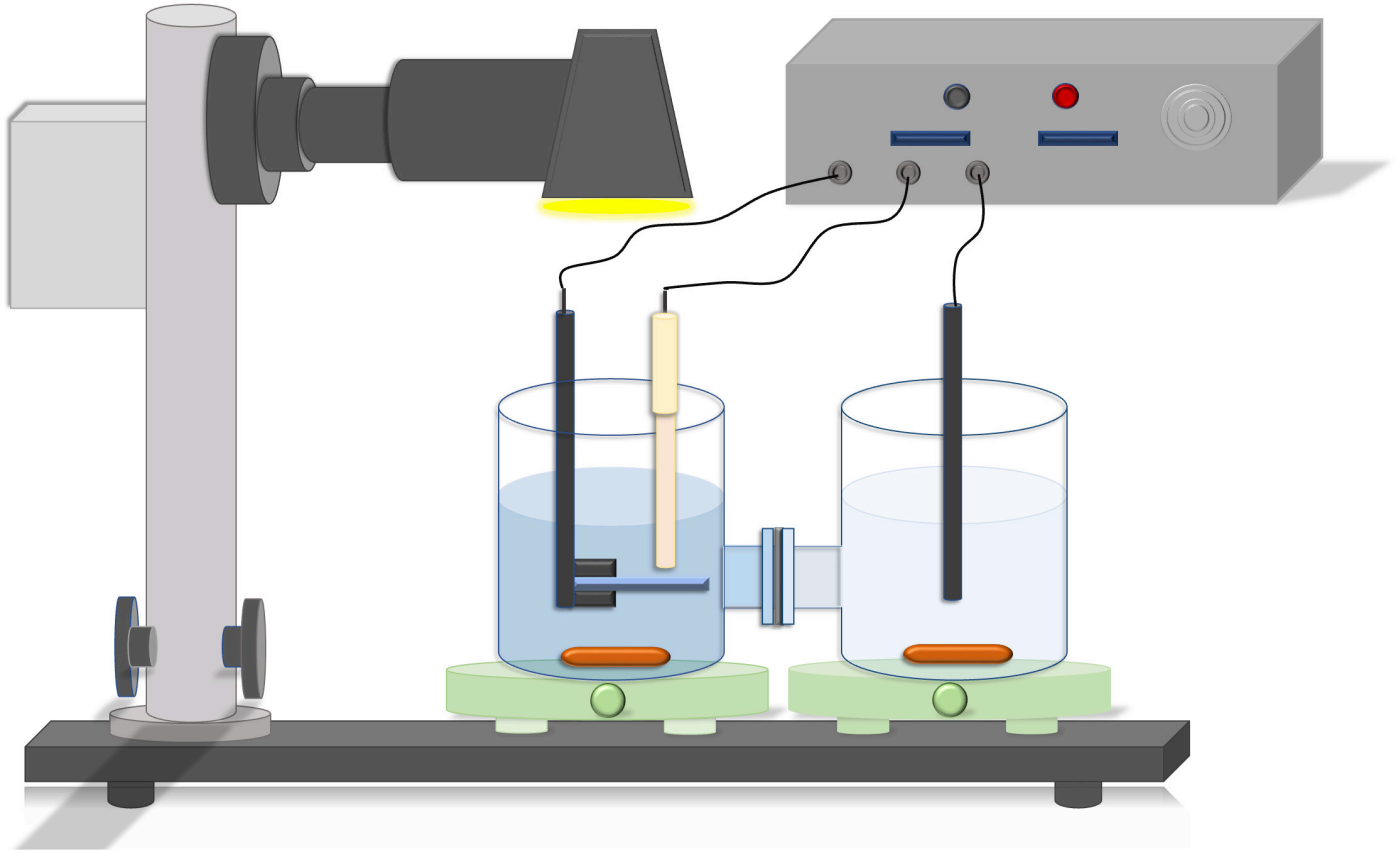
Testing setup of the photoelectrocatalytic water purification system.

The efficiency of photoelectrocatalytic organic degradation and heavy metal ion reduction in the proposed new PEC system was evaluated by degrading MB and reducing different types of heavy metal ions. The experimental setup is shown in Fig. 8. The 2 beakers of an H-type electrochemical cell contained 50 ml of 0.1 M sodium sulfate solutions with 10 μM MB and 1 ppm of different heavy metal ions, respectively. The 2 reaction chambers were separated by a proton-exchange membrane (Nafion 117, Dupont) [31]. The 2 cm × 2 cm photocatalyst electrode and an Ag/AgCl reference electrode were placed in the MB solution, while a carbon rod was placed in the heavy metal ion solution. Every 30 min, 100 μl of solution was taken out from the tube and the concentration of MB was measured using a UV/visible spectrophotometer (model SP-UV1100, DLAB Scientific Co., Ltd) at a wavelength of 664 nm. The concentration of heavy metal ions was measured by inductively coupled plasma mass spectrometry.

The performance of the highly integrated microfluidic PEC system was also evaluated by degrading MB and reducing different types of heavy metal ions. A 0.1 M sodium sulfate solution containing both 10 μM MB and 1 ppm of different heavy metal ions was injected into the microchannel at different velocities, passing through the photocatalyst electrode, the reference electrode, and the heavy metal ion reduction electrode in sequence. The solution at the outlet was collected, followed by concentration measurements of MB and heavy metal ions.

## Data Availability

All data of this study are available from the corresponding author upon request.

## References

[1] United Nations Educational, Scientific and Cultural Organization. *The United Nations world water development report 2018*. Paris (France): United Nations Educational, Scientific and Cultural Organization; 2018.

[2] United Nations Educational, Scientific and Cultural Organization. *The United Nations world water development report 2020*. Paris (France): United Nations Educational, Scientific and Cultural Organization; 2020.

[3] GBD 2013 Risk Factors Collaborators, Forouzanfar MH, Alexander L, Anderson HR, Bachman VF, Biryukov S, Brauer M, Burnett R, Casey D, Coates MM. Global, regional, and national comparative risk assessment of 79 behavioural, environmental and occupational, and metabolic risks or clusters of risks in 188 countries, 1990–2013: A systematic analysis for the Global Burden of Disease Study 2013. Lancet. 2015;386(10010):2287–2323. 26364544 10.1016/S0140-6736(15)00128-2 PMC4685753

[4] Mitra S, Chakraborty AJ, Tareq AM, Emran TB, Nainu F, Khusro A, Idris AM, Khandaker MU, Osman H, Alhumaydhi FA, et al. Impact of heavy metals on the environment and human health: Novel therapeutic insights to counter the toxicity. J King Saud Univ Sci. 2022;34(3): Article 101865.

[5] Wasewar KL, Singh S, Kansal SK. Process intensification of treatment of inorganic water pollutants. In: Devi P, Singh P, Kansal SK, editors. *Inorganic pollutants in water*. Amsterdam (the Netherlands): Elsevier; 2020. p. 245–271.

[6] Chong MN, Jin B, Chow CWK, Saint C. Recent developments in photocatalytic water treatment technology: A review. Water Res. 2010;44(10):2997–3027. 20378145 10.1016/j.watres.2010.02.039

[7] Wang Z, Wu A, Colombi Ciacchi L, Wei G. Recent advances in nanoporous membranes for water purification. Nano. 2018;8(2): Article 65. 10.3390/nano8020065 PMC5853697 29370128

[8] Karim Z, Mathew AP, Grahn M, Mouzon J, Oksman K. Nanoporous membranes with cellulose nanocrystals as functional entity in chitosan: Removal of dyes from water. Carbohydr Polym. 2014;112:668–676. 25129796 10.1016/j.carbpol.2014.06.048

[9] Pera-Titus M, García-Molina V, Baños MA, Giménez J, Espluga S. Degradation of chlorophenols by means of advanced oxidation processes: A general review. Appl Catal B Environ. 2004;47(4):219–256.

[10] Fujishima A, Zhang X, Tryk D. TiO_2_ photocatalysis and related surface phenomena. Surf Sci Rep. 2008;63(12):515–582.

[11] Cowan AJ, Tang J, Leng W, Durrant JR, Klug DR. Water splitting by nanocrystalline TiO_2_ in a complete photoelectrochemical cell exhibits efficiencies limited by charge recombination. J Phys Chem C. 2010;114(9):4208–4214.

[12] Spanhel L, Weller H, Henglein A. Photochemistry of semiconductor colloids. 22. Electron ejection from illuminated cadmium sulfide into attached titanium and zinc oxide particles. J Am Chem Soc. 1987;109(22):6632–6635.

[13] Khlyustova A, Sirotkin N, Kusova T, Kraev A, Titov V, Agafonov A. Doped TiO_2_: The effect of doping elements on photocatalytic activity. Mater Adv. 2020;1:1193–1201.

[14] Cerdán-Pasarán A, López-Luke T, Mathew X, Mathews NR. Effect of cobalt doping on the device properties of Sb_2_S_3_-sensitized TiO_2_ solar cells. Sol Energy. 2019;183:697–703.

[15] Yadav S, Jaiswar G. Review on undoped/doped TiO_2_ nanomaterial; synthesis and photocatalytic and antimicrobial activity. J Chin Chem Soc. 2017;64(2):103–116.

[16] Siriwong C, Wetchakun N, Inceesungvorn B, Channei D, Samerjai T, Phanichphant S. Doped-metal oxide nanoparticles for use as photocatalysts. Prog Cryst Growth Charact Mater. 2012;58(2–3):145–163.

[17] Sun Y-Y, Zhang S. Kinetics stabilized doping: Computational optimization of carbon-doped anatase TiO_2_ for visible-light driven water splitting. Phys Chem Chem Phys. 2016;18:2776–2783. 26725589 10.1039/c5cp07109g

[18] Tang R, Jiang Q, Liu Y. Preparation and study on photocatalytic activity of N-doped TiO_2_ decorated N-doped graphene. Procedia Eng. 2017;205:573–580.

[19] Bhanvase BA, Shende TP, Sonawane SH. A review on graphene–TiO_2_ and doped graphene–TiO_2_ nanocomposite photocatalyst for water and wastewater treatment. Environ Technol Rev. 2017;6(1):1–14.

[20] Sohn YS, Smith YR, Misra M, Subramanian VR. Electrochemically assisted photocatalytic degradation of methyl orange using anodized titanium dioxide nanotubes. Appl Catal B Environ. 2008;84(3–4):372–378.

[21] Ye S, Chen Y, Yao X, Zhang J. Simultaneous removal of organic pollutants and heavy metals in wastewater by photoelectrocatalysis: A review. Chemosphere. 2021;273: Article 128503. 33070977 10.1016/j.chemosphere.2020.128503

[22] Wang D, Chen S, Lai S, Dai W, Yang L, Deng L, Suo M, Wang X, Zou J-P, Luo S-L. Advanced municipal wastewater treatment and simultaneous energy/resource recovery via photo(electro)catalysis. Chin Chem Lett. 2023;34(5): Article 107861.

[23] Liu C, Ding Y, Wu W, Teng Y. A simple and effective strategy to fast remove chromium (VI) and organic pollutant in photoelectrocatalytic process at low voltage. J Chem Eng. 2016;306:22–30.

[24] Jeon TH, Koo MS, Kim H, Choi W. Dual-functional photocatalytic and photoelectrocatalytic systems for energy- and resource-recovering water treatment. ACS Catal. 2018;8(12):11542–11563.

[25] Zhao X, Guo L, Qu J. Photoelectrocatalytic oxidation of Cu-EDTA complex and electrodeposition recovery of Cu in a continuous tubular photoelectrochemical reactor. J Chem Eng. 2014;239:53–59.

[26] Byrne C, Fagan R, Hinder S, McCormack DE, Pillai SC. New approach of modifying the anatase to rutile transition temperature in TiO_2_ photocatalysts. RSC Adv. 2016;6:95232–95238.

[27] Raghavan N, Thangavel S, Venugopal G. Enhanced photocatalytic degradation of methylene blue by reduced graphene-oxide/titanium dioxide/zinc oxide ternary nanocomposites. Mater Sci Semicond Process. 2015;30:321–329.

[28] Zhou P, Cui T. Enhanced photocatalytic efficiency by layer-by-layer self-assembly of graphene and titanium dioxide on shrink thermoplastic film. Microsyst Technol. 2020;26:3793–3798.

[29] Hayali A, Reeves RJ, Alkaisi MM. Wavelength selective solar cells using triple cation perovskite. Nano. 2022;12(19): Article 3299. 10.3390/nano12193299 PMC9565531 36234425

[30] Zhang X, Zhang P, Zhang W, Chen J, Hu F. Preparation of UV curable optical adhesive NOA81 bionic lotus leaf structure films by nanoimprint technique and the applications on silicon solar cells. Coatings. 2023;13(5): Article 867.

[31] Lei J, Cai Q, Yang Q, Wang Y. Removal of carbon tetrachloride by enhanced reduction in a dual-chamber electrochemical reactor. J Environ Eng. 2021;147(8): Article 04021024.

